# Assessing the knowledge of Alzheimer’s disease among interns from different healthcare professions in Jeddah, Saudi Arabia: a cross-sectional study

**DOI:** 10.3389/fpubh.2025.1720277

**Published:** 2025-12-11

**Authors:** Majed Alharbi, Ahmad J. Almalki, Bayan Alghamdi, Aljoharah Almalki, Nouf Jenaideb, Shahad Khinkar, Reem Alharbi, Mohammad S. Alzahrani

**Affiliations:** 1 Department of Pharmaceutical Chemistry, Faculty of Pharmacy, King Abdulaziz University, Jeddah, Saudi Arabia; 2 King Fahd Medical Research Center, King Abdulaziz University, Jeddah, Saudi Arabia; 3 Department of Pediatrics, Ministry of National Guard Health Affairs (MNGHA), King Abdulaziz Medical City, Jeddah, Saudi Arabia; 4 Department of Clinical Pharmacy, College of Pharmacy, Taif University, Taif, Saudi Arabia

**Keywords:** Alzheimer’s disease, knowledge, health professions, future healthcare providers, Saudi Arabia

## Abstract

**Introduction:**

Alzheimer’s disease is a neurodegenerative disorder affecting memory, thinking, and daily activities. It is the most common form of dementia, and it is crucial for healthcare professionals, especially interns, to have good knowledge about it for early detection and better management. This study aimed to assess the knowledge of Alzheimer’s disease among healthcare interns in Jeddah, Saudi Arabia.

**Methods:**

A cross-sectional study was conducted using convenience sampling to evaluate the level of knowledge about Alzheimer’s disease among health professional interns in Jeddah, Saudi Arabia. Data were collected using an electronic questionnaire incorporating the Alzheimer’s Disease Knowledge Scale (ADKS), which comprises 30 true/false items covering various aspects of Alzheimer’s disease. Descriptive statistics were used to summarize participant characteristics and ADKS scores. Differences in ADKS scores across healthcare professions, gender, age groups, and other participant characteristics were assessed using independent t-tests and one-way ANOVA with Tukey’s HSD *post hoc* tests. Univariate and multivariable linear regression analyses were performed to identify predictors of ADKS scores. All analyses were conducted using SAS software version 9.4.

**Results:**

The study included 414 interns from different universities in Jeddah, Saudi Arabia. The majority of participants were women (67.6%), attended public universities (84.8%), and had a mean age of 24.1 years (SD = 2.4). The largest group of participants (28.3%) were studying medicine. The level of knowledge, as measured by the ADKS score, varied across different healthcare professions, and medical students had the highest level of knowledge (19.97 ± 3.2). Regression analysis showed that being male (B = −0.6, *p* = 0.044), having a relative with Alzheimer’s disease (B = 1.1, *p* = 0.004), and studying medicine compared to allied health sciences (B = 1.7, *p* < 0.001) were significant predictors of ADKS scores.

**Conclusion:**

The interns from various healthcare professions in Jeddah, Saudi Arabia exhibited different levels of knowledge regarding Alzheimer’s disease. The interns’ knowledge was influenced by factors such as their educational background, exposure to relevant coursework, and personal experiences. These findings emphasize the significance of incorporating extensive educational and training programs related to Alzheimer’s disease for healthcare professionals.

## 1 Introduction

Dementia is one of the most common worldwide diseases which can be defined as a group of diseases that affect memory, thinking, and daily activities (1). Dementia is not a normal part of aging, and its symptoms worsen over time (1). In 2019, an estimated 55.2 million people worldwide were living with dementia. This number is projected to increase to 78 million by 2030 and 139 million people in 2050 (2). Similarly, the number of people with dementia is projected to surge from 23 million in 2015 to around 71 million by 2050 in the Asia Pacific region (3).

Alzheimer’s disease (AD), which is the most common form of dementia, accounts for 60–70% of all cases (1). AD has been defined as a neurodegenerative disease that is characterized by slow progression along with accumulation of the amyloid-beta peptides in the affected brain area (4). AD has become a major global public health concern in the context of aging populations, significantly impacting over 130,000 individuals in Saudi Arabia alone (5). Projections of AD for 2050 indicate a substantial rise in the number of cases in Saudi Arabia, with an estimated prevalence of 855,760 cases (6). Saudi Arabia stood out among Arab countries in terms of the financial burden of AD, with the highest average value of $5 billion in dementia care costs in 2021 alone (7).

Since, approximately, 60% of dementia cases are attributed to AD, it can be estimated that the total costs related to AD in Saudi Arabia amounted to approximately $3 billion (7). Assessing this burden entails considering both direct expenses, such as medical visits and hospital admissions, as well as non-medical expenses like home healthcare and transportation (8). Additionally, indirect expenses resulting from early death, loss of productivity, and informal care contribute significantly to the overall financial impact of Alzheimer’s (9). It is worth to be noted that patients diagnosed with AD are at a higher risk of experiencing a hip fracture compared to the general population, which may result in additional medical expenses and care requirements (10).

There are various causes and factors that have been associated with the development of AD, including advanced age, gender, infections, head injuries, genetic and environmental factors (11). According to Yu et al., strong evidence was found for several causes of AD, including education, cognitive activity, high body mass index in late life, hyperhomocysteinemia, depression, stress, diabetes, head trauma, hypertension in midlife, and orthostatic hypotension (11). Furthermore, recent evidence have shown that chronic periodontitis and periodontal pathogens, which promote amyloidogenic processing of AβPP and fibrillogenesis, increase the risk of AD development, indicating that poor oral health is also a risk factor of the disease (12, 13).

A study by Nagle et al. assessed the knowledge of healthcare students about dementia which can be linked to AD, the findings showed that the average knowledge level was moderate among participants (14). However, there was a significant difference between first-year and final-year students, with final-year students scoring much higher (14). Another study conducted by Mohamed N. Al Arifi revealed differences in knowledge between various specializations within the healthcare field (15).

Understanding dementia, including AD brings benefits such as early detection, timely medical advice, improved support, better disease management, and reduced stigma of having the disease (16). This knowledge empowers individuals and caregivers, leading to informed decisions, enhanced outcomes, a supportive environment, and reduced burden on families and caregivers (16, 17). However, a lack of understanding results in limited-service utilization, delayed diagnoses, inadequate disease management, misinterpretation of behaviors, and increased caregiver stress (16).

Demographic factors such as age, gender, marital status, education level, profession, family history, and prior participation in AD training programs have been found to influence knowledge and perception of AD (18). A study conducted in Saudi Arabia found that individuals with a postgraduate academic qualification, females, older individuals, and those with relatives diagnosed with AD demonstrated higher knowledge (19).

Despite the increasing global prevalence of AD and its anticipated healthcare burden, scientific literature consistently reports persistent deficits in knowledge about AD among both healthcare providers and students. Although Al Arifi (2020) reported significant variation in AD knowledge among students from medicine, pharmacy, and dentistry programs at a Saudi university, the study was focused on a single institution (15). Accordingly, there is a need for a comprehensive, multidisciplinary study assessing AD knowledge among interns of health professions across several academic programs from multiple institutions and evaluate the impact of educational experience on their competence and readiness to identify patients with AD and provide proper care for them.

Several instruments have been developed to assess the level of knowledge in both laypeople and healthcare professionals; one of these tools is the Alzheimer’s Disease Knowledge Scale (ADKS). ADKS assesses the knowledge in seven domains, which are risk factors, assessment and diagnosis, symptoms, course, life impact, caregiving, and treatment and management (20).

The present study aims to assess the level of knowledge about AD among interns from various healthcare professions in Jeddah, Saudi Arabia, and to investigate the factors that may influence their comprehension, such as educational background, exposure to relevant coursework, and clinical experiences. By accomplishing these objectives, this study aims to contribute to a deeper understanding of AD among future healthcare professionals.

## 2 Methods

### 2.1 Study design and setting

A cross-sectional study was conducted, from October 2023 to February 2024, to assess the knowledge of Alzheimer’s disease among health professional interns in Jeddah, Saudi Arabia. Jeddah is the second-largest city in Saudi Arabia with more than 4 million residents. The city is a major port city on the Red Sea coast in western Saudi Arabia. Jeddah serves as a major logistics base and a central hub for land, sea, and air transport for the kingdom.

The study was approved by the Chair of the Research Ethics Committee in accordance with the “Implementing Regulations of the Law of Ethics of Research on Living Creatures in the Kingdom of Saudi Arabia” (Approval No. PH-1444-20).

### 2.2 Participants

Participants were recruited using convenience sampling from a total population of 2,535 interns enrolled across multiple universities in Jeddah. Inclusion criteria were: (1) current enrollment as a healthcare intern in medicine, pharmacy, dentistry, nursing, or allied health sciences; and (2) voluntary completion of the electronic questionnaire. There were no exclusion criteria beyond failure to complete the survey. A total of 414 interns met the eligibility criteria and were included in the final analysis.

### 2.3 Data collection and survey tool

Data were collected via an electronic questionnaire comprising two sections: demographic information and the Alzheimer’s Disease Knowledge Scale (ADKS). Demographic variables included age, gender, marital status, number of children, college sector (public or private), professional discipline (medicine; pharmacy; nursing; dentistry; or allied health sciences, including clinical psychology, physical therapy, and paramedics), presence of a relative with AD, and prior training or education related to Alzheimer’s.

To estimate the knowledge of AD among health professional interns, ADKS scale was chosen due to its reliability and validity (20). ADKS consists of 30 true or false questions that evaluate knowledge of AD based on current scientific understanding of the disease (20). The scale covers seven domains including risk factors, assessment and diagnosis, symptoms, disease course, life impact, caregiving, and treatment and management. The ADKS was translated into the Arabic using forward-backward translation by bilingual experts, as no Arabic version of the scale is available. A pilot test was conducted to ensure clarity and cultural relevance.

### 2.4 Statistical analysis

Responses were tabulated through the survey platform and exported for analysis. No missing data were present due to the forced-response design of the questionnaire. Descriptive statistics were used to summarize demographic characteristics and ADKS scores. Differences in ADKS scores across healthcare profession, gender, age group, and other participant characteristics were analyzed using independent t-tests and one-way ANOVA, followed by Tukey’s *post hoc* test. Univariate linear regression model was first constructed with professional discipline as the sole predictor of ADKS score. The Allied Health Sciences group was selected as the reference category because it was one of the largest subgroups and represents disciplines with comparatively less direct exposure to Alzheimer’s-specific training, making it a practical and conceptually appropriate baseline for comparison. A multivariable linear regression model was then constructed to assess the independent effects of profession along with other covariates, including age, gender, college sector, presence of a relative with Alzheimer’s, and prior training. Covariates were selected based on theoretical relevance and statistical significance (*p* < 0.10) in univariate analyses. All analyses were conducted using SAS software version 9.4, with statistical significance set at *p* < 0.05.

## 3 Results

### 3.1 Characteristics of the study participants

A total of four hundred fourteen (*N* = 414) responders completed the questionnaire out of a total population of 2,535 interns across different universities in Jeddah. Almost two-thirds of the participants were female, 67.6% (*n* = 280), while 32.4% (*n* = 140) were male. The majority of the participants were less than the age of 25, 73.2% (*n* = 303), and 26.8% (*n* = 111) were 25 or older sequentially. Most of the interns were from public universities, 84.8% (*n* = 351), and the rest were from private universities 15.2% (*n* = 63).

It was also found that 28.3% (*n* = 117) of the participants were medical students, 22.5% (*n* = 93) were from other allied health professions, 18.1% (*n* = 75) were pharmacy students, 16.2% (*n* = 67) were nursing students, and 14.9% (*n* = 62) were dentistry students. Notably, 19.1% (*n* = 79) of participants reported having a family member diagnosed with AD, while only 6.3% (*n* = 26) had received formal training specific to AD (Table 1).

**Table 1:** Descriptive characteristics of study participants.

Characteristics	*N* (%)
Age (Mean ± SD)	24.1 ± 2.4
Gender	
Males	134 (32.4%)
Females	280 (67.6%)
Marital status	
(Single)	393 (94.9%)
College/University Sector	
Private	63 (15.2%)
Public	351 (84.8%)
Professional group	
Pharmacy	75 (18.1%)
Medicine	117 (28.3%)
Dentistry	62 (14.9%)
Nursing	67 (16.2%)
Allied health sciences	93 (22.5%)
Participants with relatives suffering from AD	79 (19.1%)
Participants who had AD-specific training	26 (6.3%)

### 3.2 Knowledge of Alzheimer’s disease

**Figure 1:**
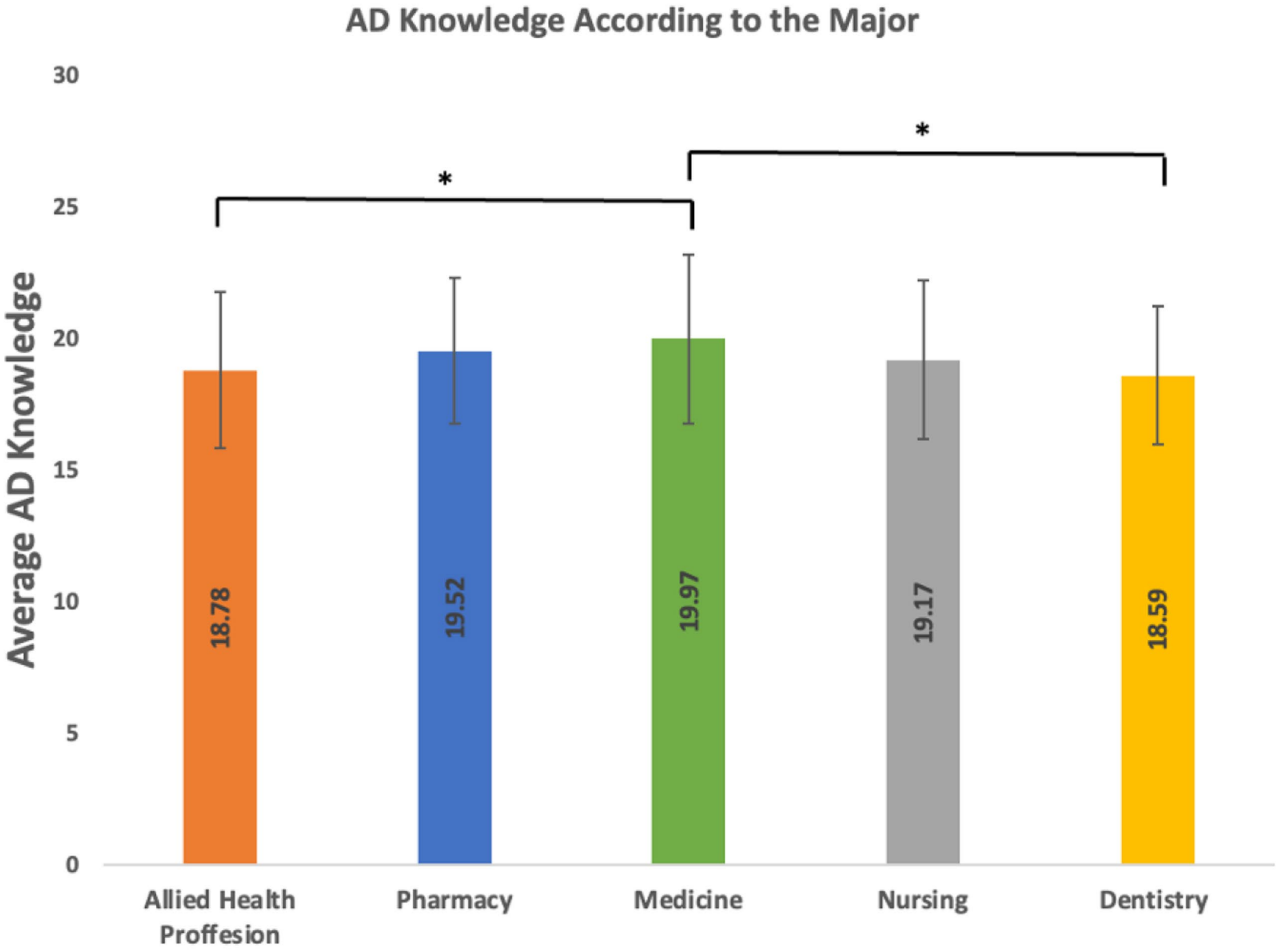
Major adjusted AD knowledge. This figure illustrates the average Alzheimer’s disease (AD) knowledge scores across five healthcare professions: Allied health, pharmacy, medicine, nursing, and dentistry (*Significant at *p* < 0.05; **Significant at *p* < 0.01).

**Figure 2:**
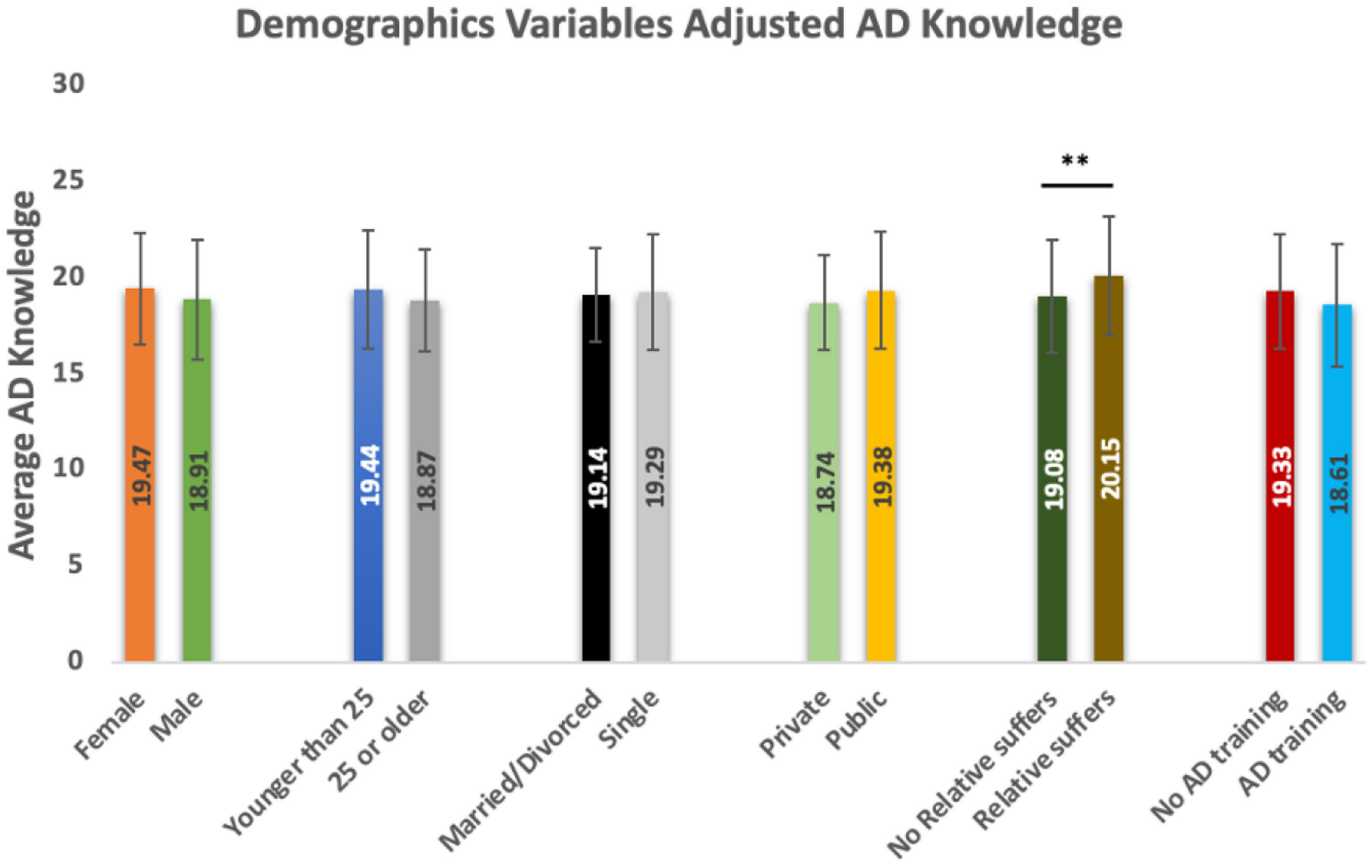
Demographics variables adjusted AD knowledge. This figure presents the average AD knowledge scores across various demographic and experiential subgroups. The *y*-axis denotes the mean AD knowledge score, while the *x*-axis categorizes participants by gender, age group, marital status, education sector, family experience, and prior AD training (*Significant at p < 0.05; **Significant at *p* < 0.01).

The mean score for the associations between intern students’ level of knowledge and demographic characteristics was 18.8 for allied health professions, 19.2 for nursing, 19.5 for pharmacy, and 18.6 for dentistry, with the highest score of 19.9 for medical interns (Table 2; Figure 1). For students who had a relative with AD, their average score was higher than those who had not. Interestingly, interns who had AD training scored less in ADKS than those who did not receive the special training (Table 2; Figure 2). With respect to educational sector, public sector participants (*M* = 19.4) reported higher knowledge than those in the private sector (*M* = 18.7). Participants with a relative suffering from AD had a notably higher mean score (*M* = 20.2) compared to those without (*M* = 19.1). Interestingly, individuals without formal AD training demonstrated a slightly higher knowledge score (*M* = 19.3) than those with training (*M* = 18.6). Across all subgroups, the variation in mean scores was relatively small, with values ranging approximately between 18.6 and 20.2.

**Table 2:** Intern students’ level of AD knowledge and demographic characteristics.

Variable	ADKS Score (Mean ± SD)	*p*-value
Gender		
Male	18.9 ± 3.2	0.073
Female	19.5 ± 2.9	
Age		
Less than 25	19.4 ± 3.1	0.086
25 years or older	18.9 ± 2.7	
Relative with AD		
Yes	20.2 ± 3.1	0.004
No	19.9 ± 2.9	
Special training in AD		
Yes	18.6 ± 3.2	0.234
No	19.3 ± 2.9	
Sector		
Public School	19.4 ± 3.1	0.069
Private School	18.7 ± 2.4	
Profession		
Allied Health Sciences	18.8 ± 2.9	0.012
Nursing	19.2 ± 3	
Pharmacy	19.5 ± 2.6	
Dentistry	18.6 ± 2.8	
Medicine	19.9 ± 3.2	

In terms of the ADKS score, the lowest score was achieved by one participant out of the total 414 participants, while only one participant, again, had the highest ADKS score. According to the findings in the total ADKS, the result shows that medical students showed greater AD knowledge than allied health professions and dentistry students, while other professions showed a similar level of knowledge to the AD.

**Figure 3:**
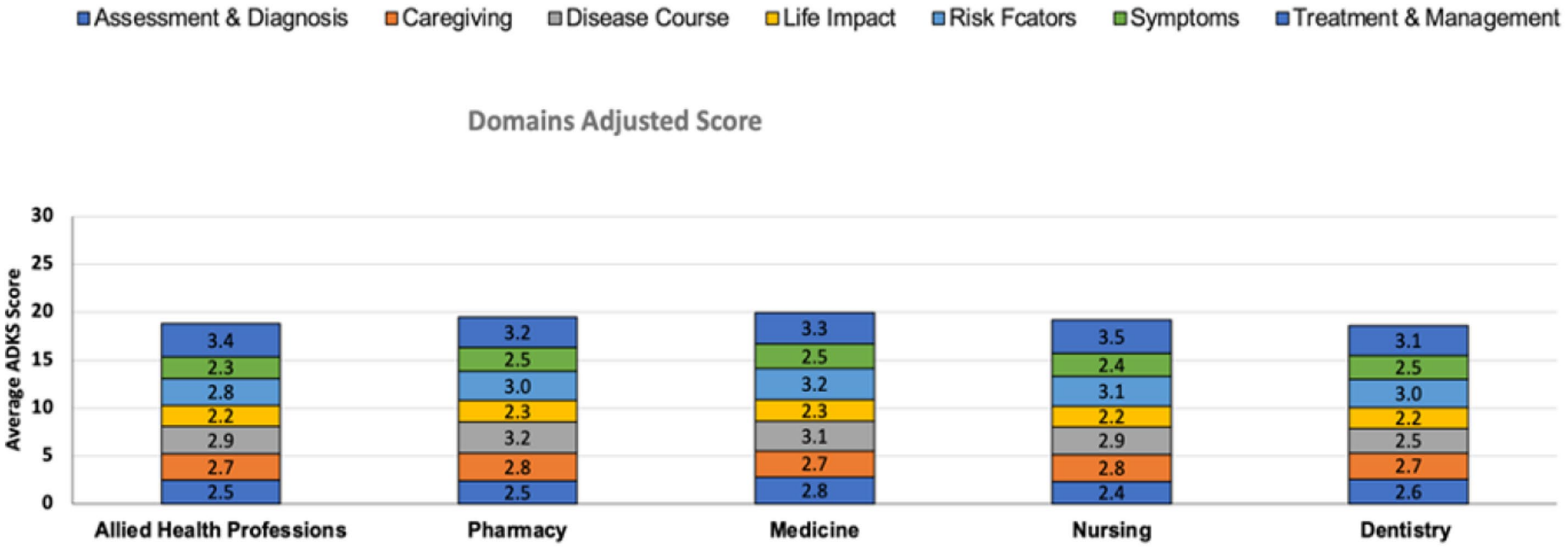
Domains adjusted ADKS score. This figure illustrates the average domain-adjusted Alzheimer’s Disease Knowledge Scale (ADKS) scores across seven domains for five academic disciplines: Allied health professions, pharmacy, medicine, nursing, and dentistry. The domains assessed include assessment & diagnosis, caregiving, disease course, life impact, risk factors, symptoms, and treatment & management. Each colored segment in the stacked bars represents the average score in a specific domain.

On the contrary, other domains of ADKS scale demonstrate different results (Table 3; Figure 3). Figure 3 shows that among all disciplines, Nursing exhibited the highest overall domain knowledge, particularly in the Assessment & Diagnosis domain (*M* = 3.5), followed by Medicine (*M* = 3.3), Allied Health Professions (*M* = 3.4), Pharmacy (*M* = 3.2), and Dentistry (*M* = 3.1). Consistently lower scores were observed in the Caregiving domain across all fields, with average scores ranging between 2.5 and 2.8. The Disease Course and Symptoms domains also showed moderate variability among disciplines. For instance, Dentistry reported the highest knowledge in Caregiving (*M* = 3.0), while Medicine and Nursing demonstrated superior scores in Symptoms and Assessment & Diagnosis, respectively.

**Table 3:** ADKS content domains and professional groups.

Domain	Items	% Correct	Mean (SD)	Professions					p-value	Significant difference
				Nursing	Medicine	Pharmacy	Dentistry	Allied health		
Life impact	3	74.4%	2.2 (0.8)	2.2 (0.8)	2.3 (0.7)	2.3 (0.8)	2.2 (0.8)	2.2 (0.8)	0.903	
Risk factors	6	50.4%	3.0 (1.2)	3.1 (1.3)	3.2 (1.2)	3.0 (1.0)	3.0 (1.3)	2.8 (1.2)	0.059	
Symptoms	4	61.1%	2.4 (0.9)	2.4 (0.9)	2.5 (0.9)	2.5 (0.9)	2.5 (0.9)	2.3 (0.8)	0.475	
Treatment and management	4	82.8%	3.3 (0.8)	3.5 (0.7)	3.3 (0.8)	3.2 (0.8)	3.1 (0.9)	3.4 (0.7)	0.047	
Assessment	4	64.9%	2.6 (0.9)	2.4 (1.0)	2.8 (0.9)	2.5 (0.9)	2.6 (1.0)	2.5 (0.9)	0.007	Medicine > Pharmacy, Nursing
Caregiving	5	54.4%	2.7 (1.0)	2.8 (0.9)	2.7 (1.0)	2.8 (1.0)	2.7 (1.0)	2.7 (1.0)	0.812	
Course of the disease	4	73.8%	3.0 (0.9)	2.9 (0.9)	3.1 (0.8)	3.2 (0.7)	2.5 (0.9)	2.9 (0.9)	0.812	Pharmacy > Allied Health, Dentistry; Medicine > Dentistry

As shown in Figure 3, in the risk factors domain, medical students in comparison with allied health professions have the highest score. Moreover, they outperformed pharmacy and nursing students in the assessment and diagnosis domain with a higher score. On the other hand, pharmacy students performed well in the course domain compared to dentistry and allied health care. Additionally, all the students achieved the same in the three remaining domains (life impacts, symptoms, and caregiving).

Table 3 shows a comparative analysis of domain-specific knowledge across five professional groups: Nursing, Medicine, Pharmacy, Dentistry, and Allied Health. The assessment encompassed seven key domains related to clinical knowledge and understanding. For each domain, the table reports the percentage of correct responses, the average score, and statistically significant interprofessional differences. Across all domains, the highest overall knowledge was observed in the Treatment and Management domain (82.8% correct), while the lowest performance was recorded in the Risk Factor domain (50.4% correct).

Analysis of variance (ANOVA) was employed to assess differences across professions. In the Life Impact domain, no significant differences were found among the professional groups (*p* = 0.903), with mean scores ranging narrowly from 2.2 to 2.3. Similarly, no significant differences were observed in the Symptoms (*p* = 0.475) and Caregiving (*p* = 0.812) domains, despite moderate variability in mean scores. Although differences in Risk Factor scores approached statistical significance (*p* = 0.059), they did not meet the threshold. The highest performance in this domain was noted among medicine and nursing professionals. Statistically significant differences were detected in three domains. In Treatment and Management (*p* = 0.047), mean scores varied modestly, with nursing professionals scoring highest (*M* = 3.5 ± 0.7). In the Assessment domain (*p* = 0.007), medicine professionals achieved significantly higher scores (*M* = 2.8 ± 0.9) compared to their counterparts in nursing and pharmacy. The most pronounced interprofessional differences were observed in the Course of the Disease domain (*p* < 0.001), where pharmacy professionals scored significantly higher than those in allied health and dentistry, and medicine professionals outperformed those in dentistry.

### 3.3 Regression models predicting knowledge of Alzheimer’s disease

Table 4 shows the results of simple and multiple linear regression models examining predictors of ADKS scores across healthcare professions and selected demographic and experiential variables. Model 1 (simple linear regression) includes only professional group predictors, while Model 2 (multiple linear regression) incorporates additional covariates such as gender, age, family and training experience with AD, and university sector.

**Table 4:** Regression models predicting knowledge of Alzheimer’s disease.

Variables	Model 1				Model 2			
	B	SE	95% CI (Lower, Upper)	*p*-value	B	SE	95% CI (Lower, Upper)	*p*-value
Intercept	18.8	0.3	18.2, 19.4	< 0.001	18.3	0.6	17.2, 19.4	< 0.001
Professional discipline*								
Nursing	0.4	0.5	−0.5, 1.3	0.406	0.4	0.5	−0.5, 1.3	0.379
Pharmacy	0.7	0.5	−0.2, 1.6	0.109	0.8	0.5	−0.1, 1.7	0.069
Dentistry	−0.2	0.5	−1.1, 0.8	0.698	0.5	0.5	−0.5, 1.5	0.361
Medicine	1.2	0.4	0.4, 2.0	0.004	1.7	0.4	0.9, 2.5	< 0.001
Male (vs Female)	–	–		–	−0.6	0.3	−1.3, −0.1	0.044
Age > 25 (vs age ≤ 25)	–	–		–	−0.7	0.4	−1.4, 0.1	0.079
Having a Relative with AD	–	–		–	1.0	0.4	0.3, 1.8	0.004
AD Training	–	–		–	−0.7	0.6	−1.8, 0.5	0.267
Public University (vs private)	–	–		–	0.5	0.5	−0.4, 1.4	0.237

^*^Allied health professions was omitted from the model and used as the reference category for professional disciplines.

In Model 1, using allied health professionals as the reference group, only the medicine group showed a statistically significant higher ADKS score, with a mean difference (B) of 1.2 (*p* = 0.004). The differences for nursing (*B* = 0.4, *p* = 0.406), pharmacy (*B* = 0.7, *p* = 0.109), and dentistry (*B* = −0.2, *p* = 0.698) were not statistically significant. The model was statistically significant overall (*p* < 0.0001), indicating meaningful differences across professions.

Model 2 adjusted for potential confounders. The difference in ADKS scores for medical interns remained statistically significant and increased slightly (*B* = 1.7, *p* < 0.001). Differences for other professional groups remained non-significant. Among the covariates, male gender was associated with a significantly lower ADKS score (*B* = −0.6, *p* = 0.045), while having a relative with AD was significantly associated with higher scores (*B* = 1.0, *p* = 0.004). No significant impacts were observed for age over 25 (*B* = −0.7, *p* = 0.079), prior training in AD (*B* = −0.7, *p* = 0.267), or attending a public university (*B* = 0.5, *p* = 0.237).

## 4 Discussion

Neurodegenerative diseases are expected to become among the top global health issues that humankind would face in the near future, and The Saudi population are expected to face a significant increase in neurodegenerative diseases including AD and dementia. The reports indicate that there will be an increase by around 900% by 2050 in these cases in the Saudi population (6). Due to the unavailability of therapeutic agents to modify the AD course, there is a vital need for a multidisciplinary approach to involve all healthcare providers in the decision-making in order to improve the quality of life for AD patients (21).

To know the ability of the different health professionals to identify AD patients, this is the first study to evaluate AD knowledge among interns from various healthcare professions across multiple universities in Jeddah, Saudi Arabia. This study evaluated the knowledge of 414 interns on AD, with an average score of 19.28 indicating moderate knowledge. In comparison, a study of healthcare staff in Queensland, Australia found a higher mean score of 23.6 on the ADKS questionnaire (16). In the current study, females and males showed similar knowledge regarding AD with mean scores of (19.47 ± 2.9058, 18.91 ± 3.1275), respectively, which match the findings of Queensland study (16). Interestingly, individuals without formal AD training demonstrated a slightly higher knowledge score than those who received formal AD specialized training, indicating that informal learning or other experiential factors may play a role in knowledge acquisition.

In addition, the findings suggest a limited prevalence of AD-related training among the study population, despite a notable proportion having personal exposure to the disease through affected relatives. In the current study, in consistence with a previous study conducted on the general population in the Aseer region of Saudi Arabia, individuals with a relative suffering from AD had higher scores compared to those who do not have family experience with the disease suggesting that personal exposure to the disease may enhance awareness about the disease (22). According to the findings in the total ADKS, the result shows that medical students showed greater AD knowledge than allied health professions and dentistry students, while other professions showed a similar level of knowledge to the AD. On the contrary, other domains of ADKS scale demonstrate different, which demonstrates similar results to a previous study published in 2020 by Mohamed N. Al Arifi (15). Additionally, pharmacy students obtained significantly higher scores in the disease course domain compared to allied health professions and dentistry (Table 3). The findings underscores the need for targeted educational programs for dentistry and allied health sciences students to improve their knowledge about the disease.

At the level of subscales, a significant difference was found between medicine with allied health professions, pharmacy and nursing, and dentistry in risk factor, assessment, and disease course domains, respectively. These consistent results can be linked to the early clinical exposure of medical students to AD patients during their studies. Furthermore, this study in parallel with what has been reported previously showed that the interns had the highest level of knowledge in the treatment subscale, followed by the knowledge about life impact, disease course, assessment, symptoms, and caregiving (23). On the other hand, the risk factor subscale demonstrated the lowest level of awareness (Table 3). The findings reveal specific areas of strength and disparity in clinical knowledge across healthcare professions, particularly highlighting medicine’s relative strength in assessment and disease course knowledge, and pharmacy’s superior understanding of disease progression. The results suggest the potential benefit of curricular alignment or targeted training interventions to address gaps in interprofessional knowledge. Overall, these results suggest that while knowledge levels are relatively comparable across disciplines, distinct domain-specific strengths and gaps exist. These insights may inform targeted educational interventions to enhance comprehensive AD knowledge across healthcare fields.

In addition, the regression analysis results suggest that professional background, particularly being in medicine, and personal experience with AD are important predictors of AD knowledge. The lack of significant impacts for prior training and university sector may reflect variations in curriculum quality or delivery across programs.

Lack of knowledge about the early symptoms of neurodegenerative diseases among health professionals presents a barrier to achieving the timely diagnosis and management of these diseases (24). This knowledge deficit about AD is a critical factor that would negatively affect the ability of health professionals to identify and diagnose people suffering from AD (24). Several reports have shown that people with mild cognitive impairment are mostly unaware of their condition, and it is important to improve the knowledge of healthcare professionals in the risk factor domain to identify the individuals at high risk of developing AD which can help in early prevention of the progression of the disease through addressing the modifiable risk factors via personalized disease prevention plans (21, 24).

Moreover, the present study revealed that interns who received specialized training on AD obtained lower score of knowledge about the disease compared to those who did not receive the special training (Table 2, Figure 2). Therefore, an audit of the content in the continuing AD education programs should be undertaken to ensure that these programs meet the requirements and adequate outcomes are achieved and reflected on the clinical practice (25).

It is important to note that the findings of this study may have some limitations. Firstly, the ADKS used in the study only evaluates knowledge related to AD and not the other forms of neurodegenerative diseases. Secondly, since the study was conducted in one city in Saudi Arabia, the findings may not be representative of the wider community in Saudi Arabia or other parts of the world. On the other hand, this study emphasizes the importance of improving knowledge about AD among interns from various health professions and the factors that influence their comprehension about the disease within the Saudi population.

## 5 Conclusion

The present study indicates that there are differences in AD knowledge among intern students in various health fields in the studied population. Medical students demonstrated the highest level of knowledge, whereas students in allied health professions and dentistry displayed the lowest score. Customized educational strategies and interprofessional teaching, which include targeted AD training programs and teamwork across different disciplines, are suggested to fill knowledge deficiencies and improve the future healthcare providers’ understanding of AD and patient treatment.

## Data Availability

The raw data supporting the conclusions of this article will be made available by the authors, without undue reservation.
